# Transcriptome and Metabolome Combined to Analyze Quinoa Grain Quality Differences of Different Colors Cultivars

**DOI:** 10.3390/ijms232112883

**Published:** 2022-10-25

**Authors:** Yongjiang Liu, Junna Liu, Li Li, Ping Zhang, Qianchao Wang, Peng Qin

**Affiliations:** College of Agronomy and Biotechnology, Yunnan Agricultural University, Kunming 650201, China

**Keywords:** quinoa, metabolome, grain difference, transcriptome, quality

## Abstract

Quinoa (*Chenopodium quinoa* Wild.) has attracted considerable attention owing to its unique nutritional, economic, and medicinal values. Meanwhile, quinoa germplasm resources and grain colors are rich and diverse. In this study, we analyzed the composition of primary and secondary metabolites and the content of the grains of four different high-yield quinoa cultivars (black, red, white, and yellow) harvested 42 days after flowering. The grains were subjected to ultra-performance liquid chromatography-tandem mass spectrometry (UPLC-MS/MS) and transcriptome sequencing to identify the differentially expressed genes and metabolites. Analysis of candidate genes regulating the metabolic differences among cultivars found that the metabolite profiles differed between white and black quinoa, and that there were also clear differences between red and yellow quinoa. It also revealed significantly altered amino acid, alkaloid, tannin, phenolic acid, and lipid profiles among the four quinoa cultivars. Six common enrichment pathways, including phenylpropane biosynthesis, amino acid biosynthesis, and ABC transporter, were common to metabolites and genes. Moreover, we identified key genes highly correlated with specific metabolites and clarified the relationship between them. Our results provide theoretical and practical references for breeding novel quinoa cultivars with superior quality, yield, and stress tolerance. Furthermore, these findings introduce an original approach of integrating genomics and transcriptomics for screening target genes that regulate the desirable traits of quinoa grain.

## 1. Introduction

Quinoa (*Chenopodium quinoa* Wild.) is a dicotyledonous plant of the Amaranthaceae family, and is native to the Andes Mountains of South America. Global production and consumption of quinoa has exponentially increased over the past few decades [1,2] and the International Year of Quinoa was celebrated in 2013. Quinoa has attracted consumer attention as a “superfood” and is known for its cold, salt, and drought resistance; it is a C3 crop and is reputed as a “golden grain” [3,4,5]. Quinoa seeds are abundant in amino acids, proteins, vitamins, dietary fiber, carbohydrates, flavonoids, phenols, and saponins [6]. Quinoa has a generally higher protein content than most other cereals and has a complete and uniform essential amino acid profile [7]. The pharmacologically active constituents of quinoa might help lower the risks of cardiovascular and neurological disease, as well as diabetes [8]. Quinoa grain is small, which is traditionally used as a grain or mixed with flour, and is a low starch food. It is used in the preparation of various breads and baked goods. However, no existing quinoa variety is suitable for noodle or pasta preparation [2,9,10]. As quinoa is highly adaptable to widely divergent environments, it is now cultivated in France, Great Britain, the Netherlands, Italy, other European countries, and the United States. Quinoa was successfully introduced to China in 1987 and large-scale quinoa cultivation has been established there. However, the main production area for quinoa remains the Andes, in South America, where a wide range of varieties, genotypes, and wild ancestors have been found during some 5000 years of cultivation [11,12]. Quinoa cultivars differ in terms of their nutritional and functional characteristics. However, continuous quinoa cultivation on the same soil eventually degrades its nutritional characteristics and reduces grain yield [13]. Quinoa is a viable and favorable cereal alternative for countries suffering from food insecurity. It may help meet the objectives of increasing high-quality food production and accommodating the nutritional needs of an expanding global population despite severe climate change. Quinoa is highly nutritional and a rich source of bioactive ingredients with strong nutraceutical and market value. Ongoing research continues to clarify the functions and mechanisms of the abundant bioactive ingredients in quinoa. Nevertheless, quinoa yield and quality are influenced by heredity, environmental conditions, and biological factors. In particular, four quinoa varieties with different colors have differences. Therefore, it is preferable to cultivate high-quality, high-yield quinoa cultivars with stable biological characteristics. In this study, we analyzed the metabolomes and transcriptomes of red, white, yellow, and black quinoa. The results of our investigations could provide a reliable basis for breeding novel quinoa cultivars—including those conducive to noodle and pasta fabrication, and facilitate a better understanding of the factors that regulate quinoa grain quality, yield, and stress tolerance.

## 2. Results

### 2.1. Grain Metabolism in Four Quinoa Cultivars

**Figure 1:**
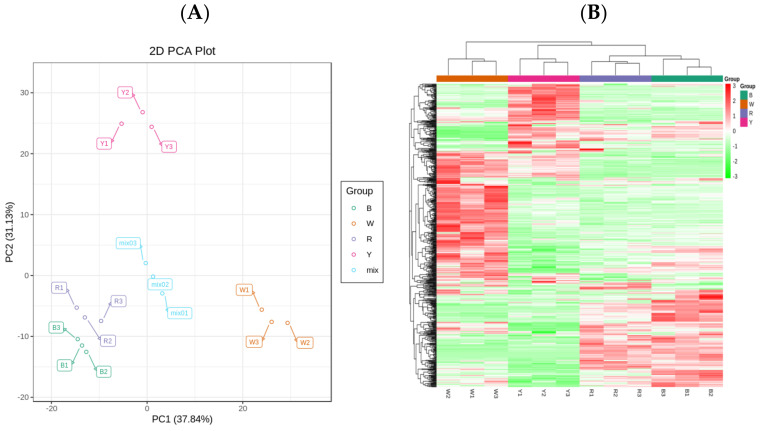
Analysis of metabolic among grains of four quinoa cultivars (**A**) total sample PCA; (**B**) clustering heatmap).

A total of 513 metabolites (Table S1) were detected in four sample groups. These included 74 amino acids and their derivatives, 82 phenolic acids, 42 nucleotides and their derivatives, 14 vitamins, 12 terpenoids, 13 tannins, 38 sugars and sugar alcohols, 38 alkaloids, 62 organic acids, and 105 lipids. There were 16 lignins and coumarins, 14 other metabolites, and three steroids. The principal component analysis (PCA) score and the heat map showed significant differences in grain metabolism among the four quinoa cultivars. The differences in grain metabolism were extensive between the white and other three quinoa cultivars, and narrow between the red and black quinoa cultivars (Figure 1).

### 2.2. Metabolic Differences among Four Quinoa Cultivars

#### 2.2.1. Analyses of Amino Acids and Their Derivatives

Table 1 shows that the differences in amino acids were smallest between B vs. R groups. They only differed in terms of 3,4-dihydroxy-L-phenylalanine and L-tyramine with log2FC for R vs. B being −7.05 and −1.69, respectively. There were obvious differences in 19 amino acids between B vs. W groups. Of these, L-threonine, L-asparagine, 3,4-dihydroxy-L-phenylalanine, and L-tyramine were downregulated and the maximum log2FC was −9.92, whereas the remaining 15 amino acids were upregulated. There were obvious differences in 19 amino acids between B vs. Y, of which L-pheny lalanyl-L-phenylalanine, homoarginine, and N-acetyl-L-phenylalanine were upregulated, whereas the other 16 amino acids were downregulated. Notably, 3,4-dihydroxy-L-phenylalanine showed the maximum difference among the groups (log2FC = 10.07). There were differences in 11 amino acids between R vs. Y. Nine of the amino acids were downregulated except homoarginine and N-acetyl-L-phenylalanine. There were differences in 20 amino acids between W vs. R. N-acetyl-L-tryptophan and L-threonine were upregulated, whereas all others were downregulated. A total of 28 amino acids differed between W vs. Y. Homoarginine, N-acetyl-L-phenylalanine, and N-acetyl-L-tryptophan were upregulated, whereas the other 25 amino acids were downregulated.

#### 2.2.2. Analyses of Phenolic Acid-Related Metabolites

Table S2 shows that 16 phenolic acids obviously differed between B vs. R groups; Syringin and feruloylmalic acid were upregulated, whereas all others were downregulated. The log2FC corresponding to p-coumaraldehyde was −16.19. A total of 35 phenolic acids obviously differed between B vs. W. A total of 23 phenolic acids, including 3,4-digalloylshikimic acid, tyrosol, 4-aminosalicylic acid, and especially p-coumaraldehyde (log2FC = −16.19), were obviously downregulated, while the other 12 phenolic acids were upregulated. A total of 38 phenolic acids obviously differed (eight upregulated and thirty downregulated) between B and Y groups. The log2FC of 2-(7-dihydroxyl)-benzofuranylferulic acid and feruloyltartaric acid were 18.23 and 18.16, respectively, and those of 4-O-(6′-O-glucosylcafeylglucosyl)-4-hydroxybenzyl alcohol and p-coumarin were −15.33 and −16.19, respectively. There were obvious differences in 39 phenolic acids between R vs. Y, of which 11 phenolic acids, including 2-(7-dihydroxyl)-benzofuranylferulic acid, salvianolic acid A, and 6′-O-ferroloyl-D-sucrose, were upregulated, while 28 phenolic acids, such as p-coumaroylcafeoyltartaric acid, tyrosol, and 4-O-(6′-O-glucosylcaffeoylglucosyl)-4-hydroxybenzoyl alcohol, were obviously downregulated. There were obvious differences in 29 phenolic acids between W vs. R groups. A total of 16 phenolic acids, such as 3,4-digalloylshikimic acid, tyrosol, and 4-aminosalicylic acid were upregulated, while 13 phenolic acids, including 4′-hydroxy-3′-methoxyacetophenone, sibiricose A5, and syringic acid-4-O-(6′-feruloyl)glucoside, were obviously downregulated. Moreover, there were obvious differences in 36 phenolic acids between W and Y groups. Of these, 12 were obviously upregulated, including 2-(7-dihydroxyl)-benzofuranylferritic acid, feruloyltartaric acid, and 3,4-diglloylshikimic acid, whereas 24 phenolic acids, such as 4-O-(6′-O-glucosylcaffeoylglucosyl)-4-hydroxybenzoyl alcohol, p-coumaroylcafeoyltartaric acid, and cimicifugic acid K, were obviously downregulated.

#### 2.2.3. Analyses of Nucleotides and Their Derivatives

A total of 42 nucleotides and their derivatives were detected in 12 samples. Table S3 shows the differences in nucleotides and their derivatives among the four quinoa cultivars. There were no differences in the nucleotides and their derivatives between B vs. R groups. However, obvious differences were observed in 11 nucleotides and their derivatives between B vs. Y groups, where N7-methylguanosine and Isoxanthopterin were obviously upregulated, whereas the other three were obviously downregulated. Seven nucleotides and their derivatives obviously differed between R vs. Y groups, and five between W and R groups, all of which were downregulated. However, the difference in xanthine was the largest (log2FC = −6.39). A total of 12 nucleotides and their derivatives differed between W vs. Y groups, and the differences were larger than those between any other group pair. Isoxanthopterin was upregulated, whereas the other 11 were downregulated.

#### 2.2.4. Analyses of Lipid-Related Metabolites

A total of 105 lipids were detected in the 12 samples. Table S4 shows the differences in lipids among different sample groups. Five lipids obviously differed between B vs. R groups; 1-O-feruloyl-3-O-caffeoylglycerol was upregulated, while 2-dodecenedioic acid, myristoleic acid, palmitoleic acid, and myristic acid were obviously downregulated. Significant differences were observed in 31 lipids between B vs. W groups. Only 1-O-Feruloyl-3-O-caffeoylglycerol was obviously downregulated (log2FC = −14.48) while the remaining 30 were upregulated. There were 33 differences in lipids between B vs. Y groups. Ricinoleic acid, eicosadienoic acid, and 12,13-epoxy-9-octadecenoic acid and 26 others were obviously upregulated, whereas myristoleic acid, 12-hydroxydodecanoic acid, and 9,12,13-trihydroxy-10,15-octadecadienoic acid were obviously downregulated. There were 33 obviously different lipids between R vs. Y groups. Of these, ricinoleic acid, eicosadienoic acid, 12,13-epoxy-9-octadecenoic acid, and 27 others were obviously upregulated, while myristoleic acid, 12-hydroxy dodecanoic acid, and 2-dodecenedioic acid were obviously downregulated. There were 38 obviously different lipids between W vs. R groups, of which only 1-O-feruloyl-3-O-caffeoylglycerol was obviously upregulated (log2FC = 15.72), while all others were obviously downregulated. There were 34 obviously different lipids between W vs. Y groups. Among them, 1-O-feruloyl-3-O-caffeoylglycerol, 12,13-epoxy-9-octadecenoic acid, ricinoleic acid, cis-10-heptadecenoic acid, and 11 others were obviously upregulated, while the rest were downregulated.

#### 2.2.5. Analyses of Organic Acid-Related Metabolites

Only L-tartaric acid obviously differed between B vs. R groups, and was down regulated. There were obvious differences in 10 organic acids between B vs. W groups. Of these, seven organic acids, including 4,8-dihydroxyquinoline-2-carboxylic acid, 2-isopropylmalic acid, and 3-isopropylmalic acid, were obviously upregulated, while L-tartaric acid, 2-hydroxy-4-methylpentanoic acid, and L-homoserine were obviously downregulated. There were obvious differences in 15 organic acids between B vs. Y groups. Among these, 4,8-dihydroxyquinoline-2-carboxylic acid and 2-furanoic acid were obviously upregulated, whereas 13-hydroxybutyric acid, D-xylonic acid, and phosphoenolpyruvate were obviously downregulated. There were obvious differences in 12 organic acids between R vs. Y groups. Among these, 4,8-dihydroxyquinoline-2-carboxylic acid, 2-hydroxyethylphosphonic acid, 2-furanoic acid, and 2-hydroxyhexanoic acid were obviously upregulated, and the rest were obviously downregulated. There were obvious differences in 17 organic acids between W vs. R groups, of which L-tartaric acid, 2-furanoic acid, 2-hydroxy-4-methylpentanoic acid, and L-homoserine were obviously upregulated, while the rest were obviously downregulated. There were obvious differences in 24 organic acids between W vs. Y groups. Among these, L-tartaric acid and 2-furanoic acid were obviously upregulated, and the rest were downregulated (Table S5).

#### 2.2.6. Analyses of Alkaloid-Related Metabolites

There were obvious differences among the four quinoa cultivars in terms of their alkaloid profiles (Table S6). The alkaloid composition and content was different between B vs. R groups. Among them, N-feruloyl-3-methoxytyramine was upregulated (log2FC = 2.02) whereas dopamine and indole-3-carboxaldehyde were downregulated (log2FC = −1.57 and −2.67, respectively). Twelve alkaloids significantly differed between B vs. W groups, of which N-hydroxytryptamine, nicotinic acid methyl ester (methyl nicotinate), and 3-hydroxypropyl palmitate glc-glucosamine were obviously upregulated, while betanin (betanidin-5-O-glucoside), dopamine, N-feruloyl-3-methoxytyramine, and indole-3-carboxaldehyde were downregulated. Eleven alkaloids obviously differed between B vs. Y groups, wherein Cimicifugamide, putrescine (log2FC = 14.50), and N-feruloyltyramine were obviously upregulated, whereas indole-3-carboxaldehyde, tryptamine, and 6-hydroxytryptamine were obviously downregulated. Ten alkaloids obviously differed between R vs. Y. Of these, four were upregulated and six were downregulated. Once again, log2FC for putrescine was 14.50. Eleven alkaloids obviously differed between W vs. R groups. Of these, betanin (betanidin-5-O-glucoside), N-feruloyl-3-methoxytyramine cimicifugamide, N-feruloyltryptamine, and dopamine were obviously upregulated, while the other five were obviously downregulated. There were 12 alkaloids that obviously differed between W vs. Y groups. Of these, cimicifugamide, indole-3-carboxylic acid, betanin (betanidin-5-O-glucoside), 5-hydroxyindole-3-acetic acid, and N-feruloyl-3-methoxytyramine were obviously upregulated, whereas the other five were obviously downregulated.

#### 2.2.7. Analyses of Sugar- and Alcohol-Related Metabolites

N-acetyl-D-glucosamine-1-phosphate and D-glucurono-6,3-lactone obviously differed between B vs. R groups, and both were upregulated. There were 12 obvious differences observed in sugars and alcohols between B vs. W groups. Of these, gluconic acid was obviously downregulated (log2FC = −1.36), while the rest were obviously upregulated. Nine metabolites obviously differed between B vs. Y groups, with D-threitol, N-acetyl-D-glucosamine-1-phosphate, isomaltulose, and melibiose being obviously upregulated, while the other five were obviously downregulated. There were eight obvious differences in sugars and alcohols between R vs. Y groups. Among these, melibiose, D-(+)-trehalose, isomaltulose, and D-threitol were obviously upregulated, whereas D-gluconic acid, D-sedoheptulose-7-phosphate, gluconic acid, and D-glucorono-6,3-lactone were obviously downregulated. There were eight differences in sugars and alcohols between W vs. R. Of these, gluconic acid was upregulated, whereas D-threonic acid, D-erythrose-4-phosphate, and D-glucosamine-1-phosphate were obviously downregulated. There were 17 obvious differences in sugars and alcohols between W vs. Y. Of these, seven were upregulated, including N-acetyl-D-glucosamine-1-phosphate, isomaltulose, and melibiose, while ten were obviously downregulated, such as D-threonic acid, D-erythrose-4-phosphate, and N-acetyl-D-mannosamine (Table S7).

#### 2.2.8. Analyses of Lignin- and Coumarin-Related Metabolites

Table S8 shows the differences in the lignin and coumarin profiles among the four quinoa cultivars. There were differences in pinoresinol-4-O-glucoside and pinoresinol-4,4′-O-di-O-glucoside between B vs. R groups with log2FC = −1.16 and −1.01, respectively. There were seven obvious differences in lignin and coumarin metabolites between B vs. W groups. Among these, pinoresinol-4,4′-O-di-O-glucoside, olivil-4,4′-di-O-glucoside, scopoletin-7-O-glucoside (scopolin), esculin (6,7-dihydroxycoumarin-6-glucoside), and syringanesinol-4′-O-glucoside were obviously upregulated, while syringanesinol and scopoletin (7-hydroxy-5-methoxycoumarin) were downregulated. There were six obvious differences in lignin and coumarin metabolites between B vs. Y groups. Among these, olivil-4,4′-di-O-glucoside, scopoletin (7-hydroxy-5-methoxycoumarin), and 5′-methoxysoliciresinol-9′-O-glucoside were obviously upregulated, whereas syringaresnol-4′-O-(6′-acetyl)glucoside, pinoresinol-4-O-(6′-acetyl)glucoside, and ayapin were obviously downregulated. There were six obvious differences in lignin and coumarin metabolites between R vs. Y groups. Of these, pinoresinol-4,4′-O-di-O-glucoside, olivil-4,4′-di-O-glucoside, scopoletin (7-hydroxy-5-methoxycoumarin), and scopoletin-7-O-gluconide were obviously upregulated, while syringaresinol-4′-O-(6′-acetyl)glucoside and pinoresinol-4-O-(6′-acetyl)glucoside were obviously downregulated. There were seven obvious differences in lignin and coumarin metabolites between W vs. R groups. Among these, syringaresinol and scopoletin (7-hydroxy-5-methoxycoumarin) were upregulated, whereas the other five were downregulated. There were five differences in lignin and coumarin metabolites between W vs. Y groups. Of these, scopoletin (7-hydroxy-5-methoxycoumarin) was upregulated, whereas pinoresinol-4-O-(6′-acetyl)glucoside, ayapin, esculin (6,7-dihydroxycoumarin-6-glucoside), and syringaresinol-4′-O-glucoside were obviously downregulated.

#### 2.2.9. Analyses of Tannin-Related Metabolites

Table 2 shows no difference in tannins between W vs. Y groups. Only 2,3-di-O-galloyl-D-glucose obviously differed between B vs. R groups (log2FC = 1.48). Eleven tannins obviously differed between B vs. W groups. Among these, 3-O-methylgallic acid was obviously upregulated, while procyanidin B3, procyanidin B2, epitheaflavic acid-3-O-gallate, procyanidin B4, and procyanidin C1 were obviously downregulated. The Log2FC for procyanidin B3 and procyanidin B2 were −20.94 and −20.87, respectively. There were obvious differences in 10 tannins between B vs. Y groups. Procyanidin B3, procyanidin B2, epitheaflavic acid-3-O-gallate, procyanidin B4, and procyanidin C1 were obviously downregulated. There were obvious differences in 12 tannins between R vs. Y groups. Among these, 1-O-galloyl-D-glucose was obviously upregulated, whereas the other 11 were obviously downregulated. Log2FC was −20.86 for both procyanidin B3 and procyanidin B2. There were obvious differences in 12 tannins between W vs. R groups. Of these, 3-O-methylgallic acid was obviously downregulated, whereas the others were obviously upregulated. Log2FC was 20.86 for both procyanidin B3 and procyanidin B2.

#### 2.2.10. Analyses of Terpenoid-Related Metabolites

A total of 12 terpenoids were detected in the 12 samples. Table S9 shows the differences in terpenoid profiles among the four quinoa cultivars. Only camaldulenic acid obviously differed between B vs. R (log2FC = −8.47) groups. There were obvious differences in six terpenoids between B vs. W groups. Of these, 24,30-dihydroxy-12(13)-enolupinol, platycogenic acid C, and 3-O-(2-O-acetylglucosyl)oleanolic acid were obviously upregulated, whereas medical acid-3-O-gluconide-28-O-rhamnosyl(1,2)-arabinoside, medical acid-3-O-glucosyl-(1,6)-glucosyl-(1,3)-glucoside, and camaldulenic acid were obviously downregulated. There were eight obvious differences in terpenoids between B vs. Y groups. Oleanolic acid-3-O-xylosyl(1→3)gluconide was obviously downregulated, whereas the other seven were obviously upregulated. Log2FC = 12.22 for oleanolic acid-3-O-gluconide. There were eight terpenoids that obviously differed between R vs. Y groups. Oleanolic acid-3-O-xylosyl(1→3)glucuronide was downregulated, while the other seven were upregulated. There were six obvious differences in terpenoids between W vs. R. Oleanolic acid-3-O-glucoside (log2FC = 12.22) and medical acid-3-O-glucoronide-28-O-rhamnosyl(1,2)-arabinoside (log2FC = 3.90) were obviously upregulated, whereas 24,30-dihydroxy-12(13)-enolupinol was obviously downregulated (log2FC = −2.67). There were nine obvious differences in terpenoids between W vs. Y groups, of which 24,30-dihydroxy-12(13)-enolupinol, pomolic acid, medical acid-3-O-glucoronide-28-O-rhamnosyl(1,2)-arabinoside, oleanolic acid-3-O-glucosyl(1→2)glucoside (log2FC = 11.74), hederagenin-3-O-glucosyl(1-2)glucosyl(1-4)arabinoside, and camaldulenic acid were obviously upregulated.

#### 2.2.11. Analyses of Vitamin-Related Metabolites

Table S10 shows the differences in vitamin profiles among the four quinoa cultivars. There were differences in two vitamins between B vs. R groups. L-ascorbic acid was upregulated (log2FC = 3.53), while biotin was obviously downregulated (log2FC = −1.02). There were five obvious differences in the vitamin content between B vs. W groups. Biotin, riboflavin, and L-ascorbic acid were upregulated, whereas nicotinamide and 4-pyridoxic acid were obviously downregulated. There were 11 obvious differences in vitamin content between B vs. Y groups. L-ascorbic acid, pyridoxal, biotin, and nicotinate-D-ribonucleoside were obviously upregulated, while pyridoxine, 4-pyridoxic acid-O-glucoside, and pyridoxine-5′-O-glucoside were obviously downregulated. There were eight obvious differences in vitamin content between R vs. Y groups. Pyridoxal, biotin, and nicotinate-D-ribonucleoside were upregulated, while pyridoxine, 4-pyridoxic acid-O-glucoside (log2FC = −10.95), and dehydroascorbic acid were obviously downregulated. There were five differences in vitamin content between W vs. R groups. Of these, 4-pyridoxic acid, pyridoxine, and 4-pyridoxic acid-O-glucoside were obviously upregulated, whereas biotin and riboflavin were downregulated. There were six obvious differences in vitamin content between W vs. Y. Nicotinate-D-ribonucleoside was obviously upregulated, and the rest were obviously downregulated. For 4-pyridoxic acid-O-glucoside, log2FC was −9.52.

#### 2.2.12. Analyses of Other Metabolites

Fourteen other metabolite classes were detected in the 12 samples. Table S11 shows the differences in other metabolite classes among the four quinoa cultivars. There were no obvious differences between B vs. R groups in terms of these metabolites. There were five obvious differences in other metabolites between B vs. W groups. Of these, 5,7-dihydroxychromone and D-(+)-melezitose-O-rhamnoside were obviously upregulated, whereas the rest were downregulated. The types and trends of six other metabolites did not obviously differ between B vs. W, or between R vs. Y groups. Among these, dihydrocharcone-4′-O-glucoside was upregulated, while 3-phospho-D-glycoric acid, N,N-dimethylformamide, palmitaldehyde, 4-methyl-5-thiazolethanol, and phenethylamine were downregulated. There were four obvious differences in other metabolites between W vs. R groups. Of these, dihydrocharcone-4′-O-glucoside and N,N-dimethylformamide were upregulated, whereas 5,7-dihydroxychromone and androsin were downregulated. There were five obvious differences in other metabolites between W vs. Y groups. Of these, dihydrocharcone-4′-O-glucoside was obviously upregulated, while 3-phospho-D-glyceric acid, 5,7-dihydroxychromone, palmitaldehyde, and phenethylamine were obviously downregulated.

### 2.3. Analyses of Correlations between Metabolome and Transcriptome among Different Quinoa Cultivars

**Figure 2:**
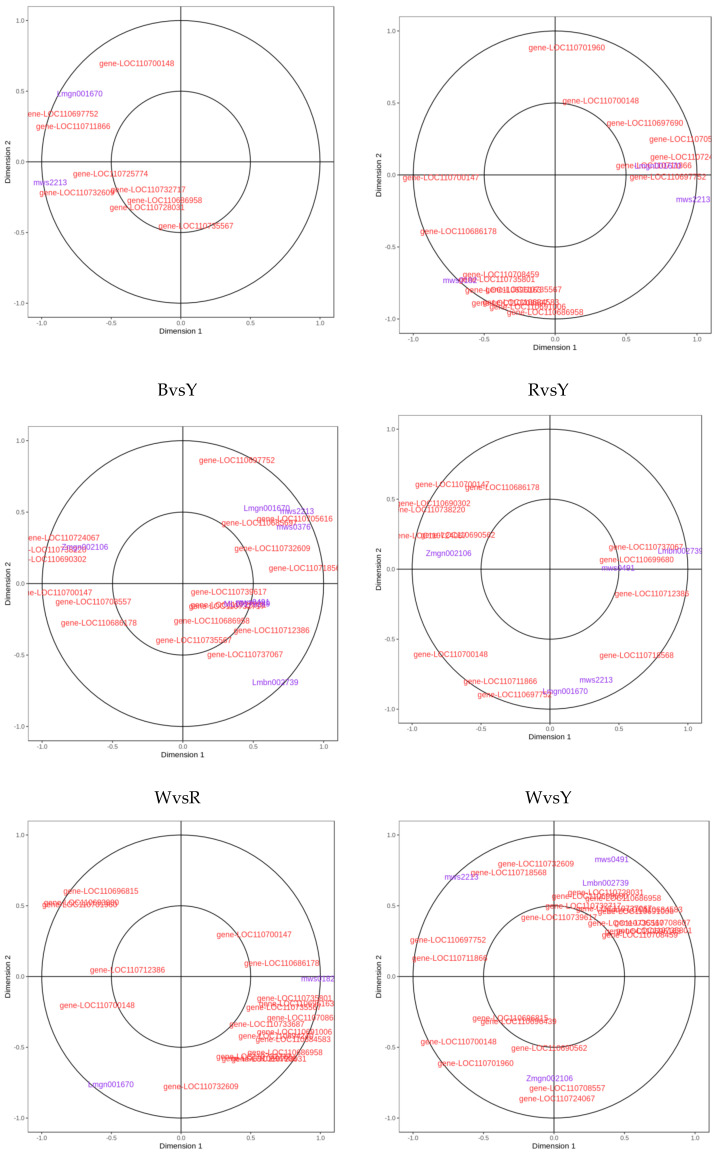
CCA results of phenylalanine metabolism pathway in different quinoa cultivars. Note: Four regions are distinguished by crosses in the figure. Within the same region, the farther away from the origin, the closer the distance to each other and the higher the correlation.

In this study, we conducted canonical-correlation analyses (CCA) (Tables S12–S23) and network correlation analyses on differentially expressed genes and metabolites in the enrichment pathways for each comparison group. The enrichment pathways common to all six comparison groups were phenylalanine metabolism, phenylpropanoid biosynthesis, and amino acid, and ABC transporter biosynthesis. According to Figure 2 and Table 3 and Table 4, thirty-eight and eight differentially expressed genes and metabolites, respectively, participated in the phenylalanine metabolic pathways of all six comparison groups. The correlation was highest between salicylic acid-2-O-glucoside and aspartate aminotransferase, cytoplasmic [EC:2.6.1.1] (*gene-LOC110705616*). Cinnamic acid had the highest correlation with amidase [EC: 3.5.1.4] (*gene-LOC110718568*). N-acetyl-L-phenylalanine had the highest correlation with enoyl-CoA hydratase [EC: 4.2.1.17] (*gene-LOC110738220*). Fumaric acid had the highest correlation with amidase [EC: 3.5.1.4] (*gene-LOC110685697*). Correlation between 3-hydroxycinnamic acid and amidase [EC: 3.5.1.4] (*gene-LOC110737067*) was the highest. Phenethylamine was highly correlated with primary amine oxidation [EC: 1.4.3.21] (*gene-LOC110732717*) and aromatic-L-amino-acid/L-tryptophan decarboxylase [EC: 4.1.1.28 4.1.1.105] (*gene-LOC110728031*). P-hydroxyphenylacetic acid had the highest correlation with amidase [EC: 3.5.1.4] (*gene-LOC110735801*). Table 3 shows that *gene-LOC110705616* expression was higher in black and red quinoa than it was in white and yellow quinoa. Table 4 shows that the relative salicylic acid content was higher in black and red quinoa than it was in white and yellow quinoa. Hence, *gene-LOC110705616* induces salicylic acid-2-O-glucoside biosynthesis. *Gene-LOC110718568* expression and relative cinnamic acid content followed the order black quinoa > red quinoa > white quinoa > yellow quinoa. Therefore, *gene-LOC110718568* promoted cinnamic acid biosynthesis, *gene-LOC110738220* inhibited N-acetyl-L-phenylalanine biosynthesis, *gene-LOC110732717* and *gene-LOC110728031* induced phenethylamine and 2-phenylethanol biosynthesis, *gene-LOC110685697* promoted fumaric acid biosynthesis, and *gene-LOC110735801* promoted p-hydroxyphenylacetic acid biosynthesis.

**Figure 3:**
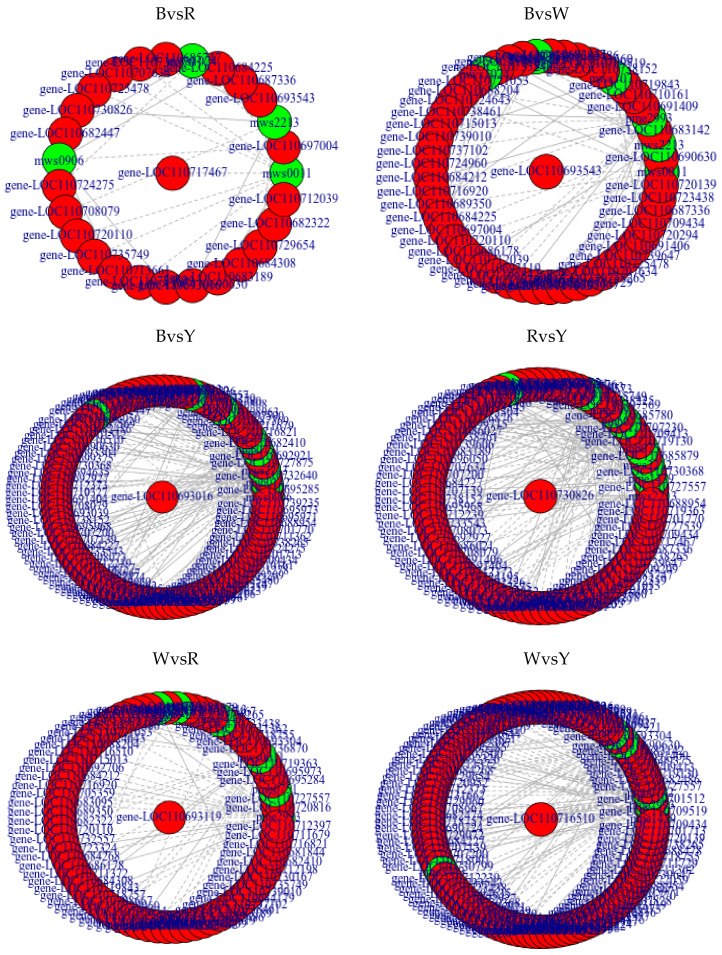
Network diagram of differential genes and metabolites among cultivars of phenylpropane biosynthesis pathway. Note: Red circles represent genes; green circles represent metabolites; solid lines are positively correlated; dotted lines are negatively correlated.

In the amino acid biosynthetic pathway, 45 and 18 differentially expressed genes and metabolites, respectively, had PCC > 0.9. Table 5 shows the differentially expressed genes that were the most highly correlated with the differentially expressed metabolites in the amino acid biosynthetic pathway. For phosphoenolpyruvate and *gene-LOC110712605*, PCC was 0.99, while for N-acetyl-L-glutamic acid, *gene-LOC110725557*, and gene-loc110683155, PCC was −0.97. Table 6 shows the differentially expressed genes and metabolites with PCC > 0.9 in the ABC transporter pathway. For D-(+)-trehalose and *gene-LOC110689873*, PCC was 0.96, whereas for lactobiose and *gene-LOC110729558*, PCC was −0.94. Figure 3 shows differentially expressed genes and metabolites with PCC > 0.8 in the phenylpropanoid biosynthetic pathway. Table S24 shows that in the phenylpropanoid biosynthetic pathway, forty and six differentially expressed genes and metabolites, respectively, had PCC > 0.9. Among these, scopoletin (7-hydroxy-5-methoxycoumarin) had the highest positive correlation with *gene-LOC110701713* (PCC = 0.93) and the highest negative correlation with *gene-LOC110715013* (PCC = −0.96). PPC was 0.9 between p-coumaric acid and *gene-LOC110724195*. For trans-5-O-(p-coumaroyl) shikimate and *gene-LOC110724195*, PCC was 0.92, whereas for trans-5-O-(p-coumaroyl)shikimate and *gene-LOC110685879*, PCC was −0.91. For caffeic aldehyde and gene-LOC110716610, PCC was 0.99, while that for caffeic aldehyde and *gene-LOC110709413* was −1.00. For cinnamic acid and *gene-LOC110685747*, PCC was 0.92, whereas for cinnamic acid and *gene-LOC110732640*, PCC was −0.92. For syringin and *gene-LOC110721053*, PCC was 0.94, while for syringin and *gene-LOC110707634*, PCC was −0.90.

## 3. Discussion

Quinoa germplasm resources and grain colors are rich and diverse. The colors of certain quinoa cultivars change during grain maturation; therefore, agronomic and quality traits widely differ among quinoa germplasms [1,14]. Meanwhile, there is broad diversity among quinoa cultivar in terms of antioxidant levels [15], nutritional characteristics [16], and dietary fiber content [17], and tannins containing good antibacterial and antioxidant effects [18]. Studies have shown that quinoa has antioxidant and bacteriostatic properties, lowers blood lipid levels, improves blood glucose, and could serve as a sustainable source of dietary supplements and functional components [19,20]. In this study, we selected seeds from three independent plants at the same growth stage and 42 d after flowering among the four cultivars (red, white, black, and yellow quinoa cultivars). After analyzing the metabolites of four different quinoa cultivars by UPLC-MS/MS, we determined the four quinoa cultivars significantly differed in terms of amino acid, alkaloid, organic acid, and tannin composition and content. Notably, they most obviously differed in terms of tannin content; the difference in tannin content was least between red and black quinoa, and the maximum tannin concentrations in red and black quinoa were far higher than those in white and yellow quinoa. The levels of the ubiquitous plant pigments procyanidin B3 and procyanidin B2 did not differ between red and black quinoa, or between white and yellow quinoa. Nevertheless, they were much higher in red and black quinoa than they were in white and yellow quinoa. Certain amino acids cannot be synthesized by the human body and must be acquired from food; the essential amino acid content is higher in quinoa than it is in rice, wheat, corn, or other cereals. Quinoa is abundant in eight amino acids essential for human health, as well as histidine required by infants and young children. Quinoa also contains lysine, threonine, and tryptophan, which are typically lacking in most other plant protein sources, and lysine is particularly deficient in cereals [21,22]. At the same time, quinoa can be made into soups, stews, biscuits, and various drinks to supplement human needs [23,24,25,26,27]. Here, we observed significant differences in amino acid and derivative composition, and content among the various quinoa cultivars. However, these differences were smallest between red and black quinoa. Our findings suggested that different quinoa cultivars will supplement different amino acids, depending on individual nutritional requirements. Moreover, the present study carefully analyzed the metabolite profiles of four different quinoa cultivars, and also compared the differences in their metabolite composition and content via metabolome and transcriptome correlation analyses. We believe that the results of the present study provide theoretical and practical references for the development and application of quinoa tannin, and that these findings lay theoretical and practical foundations for quinoa product development and enhancement, as well as the cultivation of novel quinoa cultivars with high grain yield and quality, and strong abiotic and biotic stress resistance.

## 4. Materials and Methods

### 4.1. Materials

**Figure 4:**
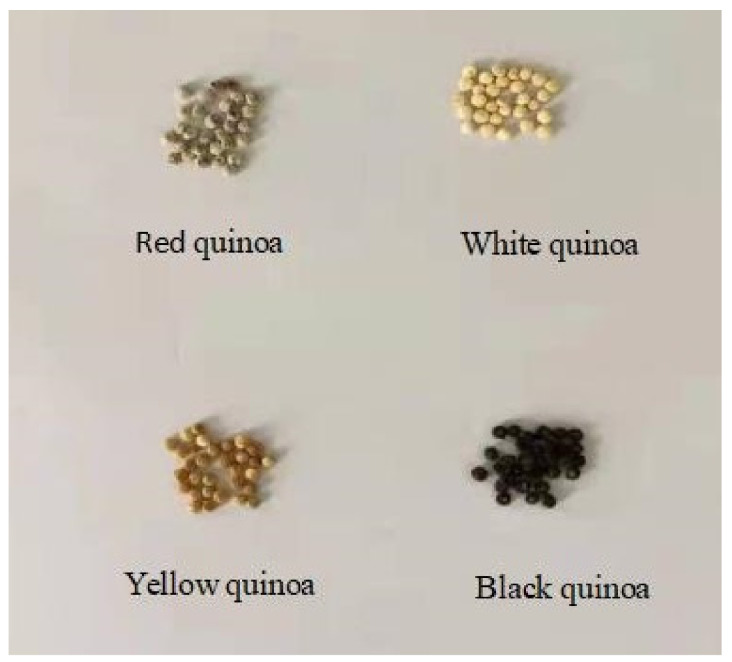
Red, white, black, and yellow quinoa cultivars (four quinoa cultivars).

Red quinoa (R), white quinoa (W), yellow quinoa (Y), and black quinoa (B) cultivars were harvested from plantations in in Xundian County, Kunming, China (102°41′ E, 25°20′ N) (Figure 4). Individual plants flowering on the same day were marked. Seeds from three independent plants at the same growth stage were selected among the four cultivars, immediately frozen in liquid nitrogen 42 d after flowering, and stored at –80 °C until later use. We allocated R, W, Y and B to red quinoa, white quinoa, yellow quinoa, and black quinoa, respectively.

### 4.2. Widely Targeted Metabolome Detection and Analysis

Biological samples were placed in a vacuum freeze-dryer (Scientz-100F, Ningbo Scientz Biotechnology Co. Ltd., Ningbo, China) and ground at 30 Hz for 1.5 min (MM 400, Retsch, Haan, Deutschland) until they were powdered. Subsequently, 100 mg powder was weighed and resuspended in 1.2 mL of 70% (*v*/*v*) methanol extract. Each sample was refrigerated at 4 °C and was rotated six times overnight to improve extraction. Each sample was centrifuged at 10,000× *g*, 4 °C, for 10 min (ANPEL, Shanghai, China, http://www.anpel.com.cn/, accessed on 23 October 2021). Each supernatant was then filtered through a 0.22-μm microporous membrane and stored in a bottle until Ultra Performance Liquid Chromatography, UPLC, (SHIMADZU Nexera X2, https://www.shimadzu.com.cn/, accessed on 23 October 2021) and Tandem mass spectrometry, MS/MS (Applied Biosystems 4500 QTRAP, http://www.appliedbiosystems.com.cn/, accessed on 23 October 2021) (UPLC-MS/MS), analysis. The chromatographic column was an Agilent SB-C18 (1.8 µm; 2.1 mm × 100 mm; Agilent Technologies, Santa Clara, CA, USA). Mobile phase A was ultrapure water with 0.1% (*v*/*v*) formic acid. Mobile phase B phase was acetonitrile with 0.1% (*v*/*v*) formic acid. The elution gradient was as follows: 0.00 min, 5% mobile phase B; linear increase in mobile phase B to 95% within 9.00 min; 95% mobile phase B for 1 min; linear decrease in mobile phase B from 10.00 min to 11.10 min; and equilibration of mobile phase B to 5% until 14 min. The flow rate was 0.35 mL/min, the column temperature was 40 °C, and the injection volume was 4 μL. The MS conditions were: electrospray ionization (ESI) temperature, 550 °C; MS voltage, 5500 V in positive mode and −4500 V in negative mode; curtain gas (CUR) pressure, 25 psi; and collision-activated dissociation (CAD) parameter set to high. For the triple quadrupole (QQQ), each ion pair was scanned and detected according to its declustering potential (DP) and collision energy (CE) [28]. Multivariate statistical analysis was used to establish a reliable mathematical model summarizing the metabolic spectrum of the research object [29]. The data were scaled by unit variance and unsupervised principal component analysis (PCA) was performed using the prcomp function in R (www.r-project.org/, accessed on 23 October 2021). An orthogonal partial least squares-discriminant analysis (OPLS-DA) model was used to analyze the metabolome data, plot score, and permutation charts for each group, and reveal the differences among groups [30]. Variable influence on projection (VIP) values were extracted from the OPLS-DA results, which included generated score and permutation graphs. Tests were run on 200 permutations to avoid overfitting. Significantly differentially expressed metabolites were identified in each group. VIP ≥ 1, Foldchange ≥ 2 and ≤ 0.5 were used to screen differentially expressed metabolites for further analysis. The metabolites identified were annotated through the Kyoto Encyclopedia of Genes and Genomes (KEGG) (http://www.kegg.jp/kegg/compound/, accessed on 4 July 2021) [31] compound database, and then mapped to the KEGG pathway database (http://www.kegg.jp/kegg/pathway.htm, accessed on 4 July 2021).

### 4.3. Transcriptome Sequencing and Data Analysis

The experimental process of transcriptome sequencing includes RNA extraction, RNA detection, library construction, and computer sequencing. Sequencing and analysis were completed by Wuhan Metware Biotechnology Co., Ltd. (Wuhan, China. https://www.metware.cn, accessed on 18 April 2021). Total RNA was extracted from seeds of three independent plants at the same growth stage, selected among the four cultivars (red, white, yellow, and black quinoa cultivars), and the RNA was analyzed by agarose gel electrophoresis in RNA detection. Following the RNA quality inspection, for completeness and to determine whether there was DNA contamination, the library construction kit (NEBNext mRNA Sample Prep Reagent Set for Illumina, San Diego, CA, USA) was used to construct a sequencing library. For the library that met the requirements, the RNA concentration was measured with high accuracy using a qubit 2.0 fluorometer (Thermo Fisher Scientific, Waltham, MA, USA), and Agilent 2100 (Agilent Technologies, Santa Clara, CA, USA) was employed to detect the insert size of the library. Finally, 12 libraries representing three repeats and four grain samples of different color lines were constructed, and the transcriptome of the library on the illuminahiseq platform sequence was tested. After the gene expression level of each sample was obtained, the differentially expressed genes between samples were analyzed. DESeq2 (https://bioconductor.org/packages/release/bioc/html/DESeq2.html, accessed on 10 November 2021) [32,33] was used for differential analysis to screen false discovery rate (FDR) < 0.05, | log2fold change | > = 1, and FDR < 0.05 was set as the threshold. After screening the differential genes according to the analysis purpose, a cluster heat map of different samples for functional annotation and enrichment analysis of differentially expressed genes, new gene analysis, variable splicing analysis, SNP, and indel analysis was produced.

### 4.4. Combined Transcriptome and Metabolome Analysis

The results of the differentially expressed metabolite (metabolome) analysis were combined with those of the transcriptome analysis. The genes showing altered transcriptomic, as well as metabolomic profiles, were mapped to the KEGG pathway chart, and histograms were plotted for them, showing the enrichment of pathways with both differential metabolites and differential genes. Correlation analyses were conducted on the differentially expressed genes and metabolites in each group. Pearson’s correlation coefficients (PCC) of the genes and metabolites were calculated using the Cor program in R (www.r-project.org/, accessed on 3 November 2021). Genes and metabolites with PCC > 0.8 were selected to plot a network diagram representing the correlations among them. The overall correlations between indicators were reflected by the output of a canonical-correlation analysis (CCA) [34].

## 5. Conclusions

In this study, we applied a widely targeted metabolomics approach to analyze the primary and secondary metabolism of various quinoa cultivars. Thus, we found the metabolite profiles differed between white and black quinoa, and there are clear differences between red and yellow quinoa. The quinoa cultivars significantly differed, mainly in terms of their amino acid, tannin, alkaloid, and phenolic acid composition and content. Six common enrichment pathways, including phenylpropane biosynthesis, amino acid biosynthesis, and ABC transporter, were common to metabolites and genes. We identified key genes highly correlated with specific metabolites, and clarified the relationship between them. The results of this study provide a reliable basis for the cultivation of novel quinoa lines with superior yield, quality, and stress resistance characteristics. Moreover, this research empirically demonstrates the successful integration of metabolomics and transcriptomics for the comprehensive analysis of the metabolite profiles of quinoa and other crops, and the identification of the genes regulating these traits. However, the transgenic road of quinoa is still a problem that requires further attention.

## Figures and Tables

**Table 1:** Analysis of differences of amino-acid-related metabolites in quinoa seeds of different cultivars.

Differential Amino Acids and Their Derivatives	Log2FC					
	BvsR	BvsW	BvsY	RvsY	WvsR	WvsY
L-threo-3-Methylaspartate	-	-	-	-	-	−1.02
N-Acetyl-L-phenylalanine	-	-	1.61	2.02	-	1.76
Pipecolic acid	-	1.68	-	-	−1.89	−1.40
3,4-Dihydroxy-L-phenylalanine	−7.05	−9.92	−10.07	−3.02	-	-
Homoarginine	-	-	1.31	1.20	-	1.11
N-Acetyl-L-glycine	-	-	−1.04	-	−1.02	−1.34
3-Hydroxy-3-methylpentane-1,5-dioic acid	-		−1.11	-	-	-
L-Homocystine	-	1.09	-	-	−1.67	-
γ-Glu-Cys	-	1.11	-	-		−1.70
L-Ornithine	-	-	-	-	−1.40	-
L-Alanine	-	-	-	-	−1.09	−1.34
L-Saccharopine	-	-	-	−1.03	-	−1.16
S-(5′-Adenosy)-L-homocysteine	-	-	-	-	-	−1.13
L-Methionine	-	-	-	-	-	−1.06
L-Tyramine	−1.69	−1.31	−1.77	-	-	-
2,6-Diaminooimelic acid	-	-	−1.57	-	-	-
N-Acetyl-L-leucine	-	-	−1.07	-	-	-
N-Acetyl-L-Glutamine	-	-	-	-	−1.06	-
L-Alanyl-L-Alanine	-	-	−1.03	−1.23	-	−1.49
N-Acetyl-L-glutamic acid	-	-	-	-	−1.14	−1.50
L-Lysine	-	1.04	-	-	−1.13	-
L-Glutamic acid	-	-	−1.34	-	-	-
L-Citrulline	-	1.19	-	-	−1.25	−1.58
L-Proline	-	-	−1.68	-	-	−2.39
L-Glutamine-O-glycoside	-	1.76	−1.89	−1.76	−1.89	−3.65
N-Acetyl-L-Tryptophan	-	-	-	-	1.02	1.81
L-Glutaminyl-L-valyl-L-valyl-L-cysteine	-	-	−1.65	−1.90	-	-
L-Aspartic acid-O-diglucoside	-	-	−2.02	−1.73	-	−2.11
L-Glycyl-L-phenylalanine	-	1.16	-	-	−1.46	−1.18
L-Glycyl-L-isoleucine	-	1.25	-	-	−1.56	−1.22
L-Alanyl-L-Phenylalanine	-	1.95	-	-	−1.75	−2.17
Oxiglutatione	-	-	-	−1.37	-	−1.95
O-Acetylserine	-	-	−2.18	−2.01	−1.16	−3.17
N-Glycyl-L-leucine	-	1.31	--	-	−1.60	−1.26
L-Phenylalanyl-L-phenylalanine	-	1.78	1.61	-	-	-
S-(Methyl)glutathione	-	-	−1.03	-	-	-
L-Threonine	-	−1.63	−1.10	-	1.03	-
L-Asparagine	-	−1.16	-	-	-	-
N-Methylglycine	-	-	−1.28	-	-	−1.11
Cycloleucine	-	1.87	-	-	−2.09	−1.58
L-Isoleucyl-L-Aspartate	-	1.48	-	-	−2.15	−2.21
L-Valyl-L-Phenylalanine	-	1.01	-	-	−1.12	-
L-Prolyl-L-Phenylalanine	-	-	-	−1.10	-	−1.22
L-Homomethionine	-	1.32	-	-	-	−1.48

Note: Log2FC is the logarithm base 2 of fold change (FC) of the differential metabolite. If log2FC is positive, it means up regulation; if log2FC is negative, it means down regulation. “-” means no difference, the same below.

**Table 2:** Analysis of differences of tannin-related metabolites in quinoa seeds among different cultivars.

Differential Tannin Species	Log2FC				
	BvsR	BvsW	BvsY	RvsY	WvsR
Gambiriin B3	-	−11.00	−11.00	−10.70	10.70
Arecatannin B1	-	−11.84	−11.84	−11.13	11.13
Procyanidin B4	-	−17.73	−17.73	−16.95	16.95
Cinnamtannin B2	-	−10.93	−10.93	−9.97	9.97
Cinnamtannin D1	-	−12.80	−12.80	−12.78	12.78
Procyanidin C1	-	−17.43	−17.43	−17.31	17.31
1-O-Galloyl-D-glucose	-	-	-	1.16	-
Procyanidin B3	-	−20.94	−20.94	−20.86	20.86
Procyanidin B2	-	−20.87	−20.87	−20.86	20.86
3-O-Methylgallic Acid	-	1.52	-	-	−1.44
Procyanidin B1	-	−12.42	−12.42	−12.07	12.07
Epitheaflavic acid-3-O-Gallate	-	−19.58	−19.58	−19.72	19.72
2,3-Di-O-galloyl-D-glucose	1.48	-	-	−1.52	1.09

**Table 3:** Enzyme point and FPKM (fragments per kilobase of transcript per million fragments mapped) value of different quinoa cultivars.

Enzyme Point	Gene ID	Gene FPKM *p* Value			
		B	R	W	Y
K14454 aspartate aminotransferase, cytoplasmic [EC:2.6.1.1]	*gene-LOC110705616*	66.89	55.32	32.20	33.49
K01692 enoyl-CoA hydratase [EC:4.2.1.17]	*gene-LOC110738220*	16.16	16.41	20.07	35.84
K01593 aromatic-L-amino-acid/L-tryptophan decarboxylase [EC:4.1.1.28 4.1.1.105]	*gene-LOC110728031*	1.94	0.97	2.94	0.93
K01426 amidase [EC:3.5.1.4]	*gene-LOC110685697*	0.10	0.02	0.02	0.00
	*gene-LOC110718568*	0.66	0.48	0.40	0.12
	*gene-LOC110735801*	6.66	9.07	18.08	6.32
	*gene-LOC110737067*	0.11	0.25	0.18	0.02
K00276 primary-amine oxidase [EC:1.4.3.21]	*gene-LOC110732717*	17.69	8.78	25.55	8.83

**Table 4:** Relative contents of different metabolites in grains of different cultivars of quinoa.

ID of Metabolites	Name of Different Metabolites	Relative Content of Metabolites			
		B	R	W	Y
Zmgn002106	N-Acetyl-L-phenylalanine	443363.33	334590.00	399070.00	1355836.67
pmb2620	Cinnamic acid	60389.00	23857.33	17504.33	4122.60
mws0491	Phenethylamine	1252800.00	890490.00	1601766.67	409326.67
mws0376	Fumaric acid	284246.67	155803.33	161770.00	83921.67
mws0182	p-Hydroxyphenyl acetic acid	1349833.33	1604800.00	3897533.33	2410566.67
ML10179289	2-Phenylethanol	136783.33	112217.33	179213.33	57010.67
Lmgn001670	Salicylic acid-2-O-glucoside	1586490.00	383650.00	22707.00	19206.00
Lmbn002739	3-Hydroxycinnamic Acid	392796.67	676676.67	640516.67	99752.67

**Table 5:** Correlation between differential genes and differential metabolites in amino acid biosynthesis pathway.

The Name of Metabolites	Gene Name	PCC
D-Erythrose-4-phosphate	*gene-LOC110732956*	−0.9
	*gene-LOC110694674*	−0.9
Isocitric Acid	*gene-LOC110697015*	0.92
	*gene-LOC110735050*	−0.92
	*gene-LOC110683155*	−0.92
3-Phospho-D-glyceric acid	*gene-LOC110702638*	0.97
	*gene-LOC110735050*	−0.94
L-Methionine	*gene-LOC110709420*	0.93
	*gene-LOC110708868*	−0.92
2,6-Diaminooimelic acid	*gene-LOC110719991*	0.93
	*gene-LOC110688007*	−0.92
N-Acetyl-L-glutamic acid	*gene-LOC110726706*	0.95
	*gene-LOC110725557*	−0.97
	*gene-LOC110683155*	−0.97
L-Glutamic acid	*gene-LOC110712605*	0.93
	*gene-LOC110688007*	−0.91
L-Citrulline	*gene-LOC110725557*	−0.91
	*gene-LOC110688707*	−0.94
L-Proline	*gene-LOC110682443*	0.91
2-Isopropylmalic Acid	*gene-LOC110709420*	0.94
	*gene-LOC110683155*	−0.92
	*gene-LOC110725557*	−0.92
Phosphoenolpyruvate	*gene-LOC110712605*	0.99
	*gene-LOC110688007*	−0.94
Anthranilic Acid	*gene-LOC110732372*	0.9
	*gene-LOC110725557*	−0.9
	*gene-LOC110683155*	−0.9
O-Acetylserine	*gene-LOC110704567*	0.94
	*gene-LOC110735050*	−0.92
3-Methyl-2-Oxobutanoic acid	*gene-LOC110683275*	0.92
	*gene-LOC110693518*	0.92
	*gene-LOC110683155*	−0.95
L-Homoserine	*gene-LOC110708482*	0.91
Citric Acid	*gene-LOC110726706*	0.9
	*gene-LOC110683155*	−0.9
L-Asparagine	*gene-LOC110705616*	0.93
3-Isopropylmalic Acid	*gene-LOC110726706*	0.93
	*gene-LOC110709420*	0.93
	*gene-LOC110725557*	−0.93

Note: PCC indicates Pearson’s correlation coefficient.

**Table 6:** Correlation between differential genes and differential metabolites in the ABC transporter pathway.

The Name of Metabolites	Gene Name	PCC
Lactobiose	*gene-LOC110689873*	0.92
	*gene-LOC110729558*	−0.94
Melibiose	*gene-LOC110683754*	−0.9
	*gene-LOC110717984*	−0.9
	*gene-LOC110729558*	−0.94
Inositol	*gene-LOC110710278*	0.91
	*gene-LOC110717984*	−0.91
L-Alanine	*gene-LOC110681663*	0.9
D-Glucose	*gene-LOC110700561*	0.9
D-Ribose	*gene-LOC110700561*	0.91
D-(+)-Trehalose	*gene-LOC110689873*	0.96
	*gene-LOC110729522*	0.92
	*gene-LOC110729558*	−0.9
	*gene-LOC110722638*	−0.92

## Data Availability

Not applicable.

## Supplementary Materials

The supporting information can be downloaded at: https://www.mdpi.com/article/10.3390/ijms232112883/s1. Reference [35] is cited in the Supplementary Materials.
