# Supplementary material for: Transcriptome and Metabolome Combined to Analyze Quinoa Grain Quality Differences of Different Colors Cultivars
Source: Int J Mol Sci. 2022 Oct 25;23(21):12883. doi: 10.3390/ijms232112883 (PMC9656266; doi:10.3390/ijms232112883)
Supplement: Supplementary file 1 [file ijms-23-12883-s001.zip › Situation description.pdf]

Situation description

The materials and methods in this article are similar to “Yongjiang Liu, Junna Liu, et al. Transcriptomics and metabolomics analyses of the mechanism of flavor synthesis in seeds of differently colored quinoa strains Genomics, (2022) ” [35] uses same batch of materials and different parts of the same set of transcriptome data. At present, this article is in nov-23-2021, genomics completed accept,It is hereby explained!
